# Liposome-loaded phage cocktail: a promising therapeutic option against postsurgical wound infections – a critical appraisal

**DOI:** 10.1097/MS9.0000000000002275

**Published:** 2024-06-17

**Authors:** Maheswaran Easwaran, Nageshwari Raja, Muthupandian Saravanan, Melaku Ashagrie Belete

**Affiliations:** a Department of Research Analytics, Saveetha Dental College and Hospitals, Saveetha Institute of Medical and Technical Sciences (SIMATS), Saveetha University, Chennai, Tamil Nadu, India; b AMR and Nanotherapeutics Laboratory, Department of Pharmacology, Saveetha Dental College and Hospitals, Saveetha Institute of Medical and Technical Sciences (SIMATS), Saveetha University, Chennai, Tamil Nadu, India; c Department of Biotechnology, Sethu Institute of Technology, Virudhunagar, Tamil Nadu, India; d Department of Medical Laboratory Science, College of Medicine and Health Sciences, Wollo University, Dessie, Ethiopia

Postoperative-surgical wound infection (P-SWI) is a serious and increasingly common complication that can occur within a month of surgery^1^. Exogenous or endogenous microbes (>10^4^) can cause severe P-SWI, leading to lethal infections such as cellulitis, abscess, osteomyelitis and bacteremia with sepsis of the skin, tissues, and organs^2^. *Mycobacterium tuberculosis*, for instance, can cause psoas abscess as a complication of spinal tuberculosis. This can lead to symptoms such as fever (around 38.5°C), hypotension (90/60 mmHg), increased leukocyte count (18 000/μl), and possibly mild anemia (8 g/dl)^3^. In ICUs, the major wound colonizing bacterial pathogens with virulence factors such as *Acinetobacter baumannii*, *Escherichia coli*, *Pseudomonas aeruginosa*, *Staphylococcus aureus*, and *Klebsiella pneumoniae* are responsible for around 63% of surgical site infections. Particularly, methicillin-resistant *Staphylococcus aureus* ^4^, *Pseudomonas aeruginosa* ^5^, and *Acinetobacter baumannii* ^6^ are the most predominant bacterial pathogens associated with extrinsic P-SWI (74%), Intrinsic P-SWI (20%), and organ or space P-SWI (6%) in hospitalized patients. These pathogens exhibit high resistance to standard antibiotics such as cefazolin, cefoxitin, cefuroxime, metronidazole, sulbactam, and vancomycin. This resistance contributes to chronic, nonhealing wounds and extensive tissue damage, leading to significantly increased patient morbidity and mortality^7^. In addition, patients with diabetes, obesity, hypovolemia, malnutrition, and weakened immune systems are more susceptible to wound complications and surgical site infections, leading to difficult-to-treat infectious agents. Alternative approaches are needed to fully evaluate the long-term efficacy, safety, and potential to reduce antibiotic resistance in different clinical settings^7^. Therefore, novel phage therapy to combat P-SWI has emerged over the last decades. Bacteriophages (phages) are a promising tool to address the challenges posed by antibiotic-resistant infectious agents as they possess unique properties, such as high bacterial lytic efficacy, self-replication, and high target specificity. In P-SWI, biofilm-forming pathogens often exhibit significantly increased antibiotic tolerance, which is a major challenge due to their complex structure and sophisticated defense mechanisms that enable them to evade conventional antibiotic and single-phage therapies.

To overcome the emerging antibiotic resistance, liposome-associated phages have gained much attention due to their potential therapeutic applications. Liposomes, like biological membranes, are self-assembling vesicles composed of phospholipids. They can be developed as carriers for therapeutic agents such as phages and offer several advantages such as functional mechanism, structure, controlled release, and enhanced skin barrier penetration, making them potentially effective for the treatment of P-SWI^8^. Phage cocktails (PCTs) hold promise as a more effective strategy for P-SWI due to broader strain targeting by specific phage tail fiber, reduce bacteria faster through a constant phage lytic sequence, and potential wound healing benefits, while their unique properties, such as persistence, safety for immunocompromised patients, and site-specific accumulation effectively address the challenges of biofilm-associated P-SWI. Liposome-shielded phage cocktail (L-PCT) systems, utilizing smaller liposomes for efficient phage encapsulation, promise enhanced phage stability, retention, and effectiveness in acidic wound environments, potentially improving overall therapeutic efficiency (87%) compared to traditional PCT therapy^8^.

L-PCT system releases phages on the skin surface through an intracellular and intercellular delivery system of liposomes. Liposomes can cover the wound surface, sustain moist conditions, and assist wound healing process^9^, potentially lead to enhanced phage retention on the bacterial biofilm. Moreover, liposomes are widely used as carriers for the delivery of mRNA vaccines and the development of a more efficient drug delivery system to humans^10^. Therefore, the L-PCT system could overcome the side effects if it can be used for drug delivery. In brief, the exposed PCTs can potentially target antimicrobial-resistant pathogens through various bacterial clearance mechanisms, including the interaction of phage tail fibers and host receptors, lytic and lysogenic patterns. On the one hand, phage-tagged bacteria have the potential to be recognized faster by dendritic or macrophage cells due to phage-mediated opsonization, which in turn enables improved phagocytic uptake. On the other hand, phages can enhance the immune response through bacterial remnants such as cell membranes and cytosolic proteins that adhere to the phage. In addition, a possible synergy between neutrophils and phages may play a key role in the elimination of phage-tolerant bacteria. Mild inflammation and rapid re-epithelialization have been shown to occur due to the action of L-PCT following the clearance of bacteria or bacterial components^11^. Few phages do not directly involve intracellular bacteria lysis but can indirectly contribute to the elimination of bacteria by interacting with phagocytes in various ways, for example, modulating ROS production, reducing ROS-induced cell damage and supporting phagocytosis. For example, the tail adhesin gp12 of the T4 phage could reduce ROS production in activated polymorphonuclear leukocytes when the phage interacts with the bacterial host and affect eukaryotic cell function and anti-inflammatory properties^11^. During phage-host infection, the phage can inhibit T cell activation and causes downregulation of chemokines (CXCL-5 and CXCL-12a), cytokines (IL-6; IL-8 and TNF-α), proinflammatory cytokines (IL-1β, NF-κB) and Toll-like receptor 4. In addition, it induces the upregulation of IL-1 receptor antagonist, IL-10 and suppressor of cytokine signaling 3, which leads to a reduction in inflammation^12,13^ (Fig. 1). Ultimately, liposomes have the potential to target specific cells with targeted administration, stabilize vaccine components, and enhance the immune response^14^. Similarly, L-PCTs also have the potential to serve as a universal platform for vaccine development. This paves the way for promising applications in immunomodulation, especially for the treatment of autoimmune and inflammatory diseases. Their programmable nature makes them attractive options as biological agents and tools for developing new forms of pharmacological medicine. Furthermore, L-PCT alongside traditional antibiotics holds promise for mitigating the emergence of multidrug-resistant bacteria, especially in patients with postoperative wound infections. L-PCT warrants further investigation as a novel therapeutic approach in the future.

**Figure 1:**
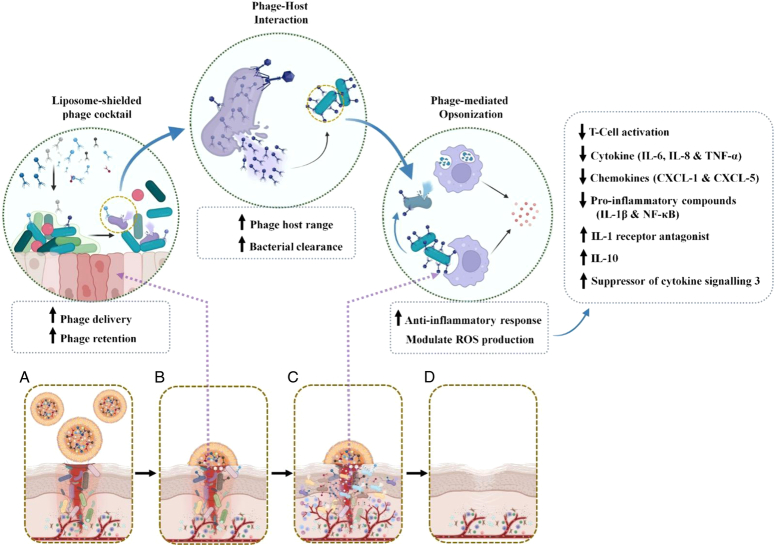
Exploring the potential of liposomal phage cocktails for clearing multidrug-resistant bacteria in postoperative surgical wound infections. (A). The diagram illustrates a surgical wound after an operation, potentially harboring harmful MDR bacteria; Liposome-shielded phage cocktail (L-PCT) are delivered as a spray directly onto the wound. Liposomes act as protective carriers, shielding the phages from harsh environments and enhancing their target bacterial cells; (B). Liposomes facilitate the internalization of phages into infected bacterial cells, ensuring effective delivery and maximizing their impact; (C). Once inside the bacteria, phages replicate, leading to the lysis (bursting) of infected cells and the release of new phage particles. Additionally, the presence of phages can stimulate the immune response to further combat the infection; (D). With the elimination of MDR bacteria, the wound can heal effectively, reducing the risk of complications and promoting patient recovery.

## Ethical approval

Not applicable.

## Consent

Not applicable.

## Source of funding

Not applicable.

## Author contribution

M.E. and N.R.: investigation, writing – original draft preparation, conceptualization, and visualization; M.S. and M.A.B.: conceptualization, writing – reviewing and editing, and supervision.

## Conflicts of interest disclosure

The authors declare no conflicts of interest.

## Research registration unique identifying number (UIN)

Not applicable.

## Guarantor

Melaku Ashagrie Belete.

## Data availability statement

All data are available in the manuscript.

## Provenance and peer review

Not applicable.
